# Commentary: Is there a minimum effective dose for vascular occlusion during blood flow restriction training?

**DOI:** 10.3389/fphys.2023.1279435

**Published:** 2023-10-02

**Authors:** Pat R. Vehrs, A. Wayne Johnson

**Affiliations:** Department of Exercise Sciences, Brigham Young University, Provo, UT, United States

**Keywords:** blood flow restriction, blood flow restriction training, KAATSU, strength training, arterial occlusion pressure

## Introduction

We read with interest the article by Das and Patons (2022) in which the authors performed a meta-analysis of 21 blood flow restriction (BFR) training studies to determine the minimal and optimal combination of resistance and occlusion pressure that resulted in strength gains. The authors report that the occlusion pressures used in the 21 studies ranged from 40% to 150% of the limb occlusion pressure (LOP). This commentary discusses the findings reported by the authors about the occlusion pressures used in their analysis of the training studies.

## Discussion

Our concerns relate to the accuracy of the information presented in Table 1 of the Das and Patons (2022) paper which refers to five studies (Laurentino et al., 2008; Manimmanakorn et al., 2013; Cook et al., 2017; Biazon et al., 2019; de Lemos Muller et al., 2019) that used occlusion pressures of 100%–150% LOP during BFR training. A careful review of these five studies suggests that the information presented by Das and Patons (2022) is inaccurate. We briefly summarize the occlusion pressures reported in each of the five studies.

Although Biazon et al. (2019) clearly indicate that the occlusion pressure “used during the BFR protocols was set at 60% of occlusion pressure,” Figure 11 in the Supplementary Material of the Das and Patons (2022) study erroneously lists the occlusion pressure used in the study as 100% LOP.

Cook et al. (2017) used a cuff pressure representing ×1.5 brachial artery systolic blood pressure (SBP) to restrict blood flow at the thigh. The authors did not actually measure the LOP of the thigh, and therefore, it is not possible to determine the relative occlusion pressure (%LOP). Figure 11 in the Supplementary Material of the Das and Patons (2022) study lists the %LOP of this paper as 150%, suggesting the erroneous interpretation of the authors that the ×1.5 brachial artery SBP was equivalent to 150% of the LOP of the thigh.

In the de Lemos Muller et al. (2019) study, the authors performed BFR of the upper arm and the upper thigh. The cuff pressure used on the upper arm was set at 20 mmHg below the brachial artery SBP, and the cuff pressure used on the upper thigh was set at 20 mmHg above the brachial artery SBP. The authors described the “partial occlusion of the limbs,” and the use of a pulse oximeter to ensure that “there was no complete interruption of blood flow.” If a pulse was not detected during BFR, the cuff pressure was reduced until the pulse was detected. In this study, it is clear that sub-occlusive cuff pressures were used. Because the LOPs of the arm and leg were not actually measured, it is not possible to determine the %LOP used in the study.

Laurentino et al. (2008) applied BFR to the upper thigh. Although the authors measured LOP of the upper thigh, they did not report actual LOP values and merely reported that the cuff pressures used to restrict blood flow in the two intervention groups averaged 125 mmHg and 131 mmHg. Given the large circumference of the upper thigh of men, it is highly unlikely that these cuff pressures represent 100% LOP of the thighs. The authors state in the Abstract that “blood flow was reduced during the exercise” (not occluded). Because the authors do not report the LOP, it is not possible to determine the %LOP. Nevertheless, Figure 11 in the Supplementary Material of the Das and Patons (2022) paper lists the %LOP as 100%.

Manimmanakorn et al. (2013) stated that during training, the KAATSU cuffs were inflated to pressures of 160 mmHg on Day 1 and were increased by 10 mmHg each day until Day 8 (and thereafter) when the cuff pressure was 230 mmHg. KAATSU cuffs are soft, multi-chambered cuffs, which are unlikely to achieve complete arterial occlusion in the upper thighs—especially at pressures between 160 mmHg and 230 mmHg. In addition, the authors do not report measuring LOP, so it is not possible to report the relative occlusion pressure (%LOP) used during training. Nevertheless, Figure 11 in the Supplementary Material of the Das and Patons (2022) study lists the cuff pressures used in the study as 100% LOP.

After reviewing the five studies referenced, we do not find support for the use of blood flow restriction pressures of 100%–150% of LOP during BFR training as reported by Das and Patons (2022). The measurement of LOP and use of occlusion pressures during training representing a %LOP were included in the criteria used by the authors to select training studies for their meta-analysis. Based on our review of the references cited in their paper, there were no studies that used blood flow restriction pressures of 100%–150% of LOP during BFR training. In four of the five studies reported by Das and Patons (2022) as using occlusion pressures of 100%–150% of LOP, it is not possible to determine the %LOP used for BFR. Based on the criteria set by the authors, these four studies should be excluded from the meta-analysis. The fifth study clearly states using an occlusion pressure of 60% LOP (not 100%–150% of LOP). These data affect the analysis of the data, discussion about the outcomes of the referenced studies, and the author’s conclusions. Mistakenly reporting studies using pressures 100%–150% of LOP can also be misleading to the readership and may have serious implications. Readers may interpret this information as suggestive that cuff pressures exceeding LOP are used (or are recommended) during BFR training. It is important that the occlusion pressure used during BFR training only partially restricts arterial blood flow into the muscle (Iida et al., 2005; Iida et al., 2007). When using pneumatic cuffs, setting the cuff pressure relative (40%–80%) to the arterial occlusion pressure (AOP) or LOP is recommended during BFR training (Scott et al., 2015; McEwen et al., 2019; Patterson et al., 2019). Using this individualized approach can attenuate the discomfort during BFR training (Spitz et al., 2022) and minimize potential risks associated with BFR training (Scott et al., 2015; Patterson et al., 2019; Anderson et al., 2022).
